# Treatment Response Trajectories of Epipharyngeal Abrasive Therapy (EAT) in Chronic Nasopharyngitis: Phenotypic Classification Into Early, Delayed, and Non-Responders Based on ΔEAT With Outcome Stratification

**DOI:** 10.7759/cureus.109478

**Published:** 2026-05-23

**Authors:** Ito Hirobumi

**Affiliations:** 1 Otolaryngology, Ito ENT Clinic, Funabashi, JPN

**Keywords:** chronic nasopharyngitis, delayed responder, early responder, epipharyngeal abrasive therapy (eat), non-responder, outcome stratification, response trajectory, temporal heterogeneity, treatment intensity, δeat

## Abstract

Background

Treatment responses to epipharyngeal abrasive therapy (EAT) for chronic nasopharyngitis are heterogeneous. Previous analyses of this cohort examined longitudinal changes in EAT and bleeding scores with stratification by tissue type and baseline severity. However, those analyses mainly evaluated overall score changes and baseline-based stratification rather than the timing of individual improvement. The novelty of the present study lies in its focus on temporal response trajectories, specifically when patients improve after EAT. This study classified patients into early responders, delayed responders, and non-responders based on changes in EAT scores during the first three months and examined their association with final outcomes.

Methods

This single-center retrospective observational study included 546 patients (100.0%) with chronic nasopharyngitis treated with EAT between August 2018 and August 2019. The primary analysis included 238 patients (43.6%) with complete EAT scores at baseline, one month, two months, and three months. Patients were classified into three temporal phenotypes based on ΔEAT, namely early responders, delayed responders, and non-responders. Final outcomes were categorized as complete resolution, partial resolution, dropout, or indeterminate. Logistic regression was performed using complete resolution as the dependent variable, with adjustment for baseline characteristics and treatment intensity.

Results

Among the 238 patients (43.6%), 103 (43.3%) were early responders, 83 (34.9%) were delayed responders, and 52 (21.8%) were non-responders. Baseline EAT scores differed significantly among the groups (one-way ANOVA, F = 27.04, p < 0.001), as did baseline bleeding scores (one-way ANOVA, F = 4.94, p = 0.008). Final outcome distribution did not differ significantly among the threshold-based response groups (χ² = 0.92, p = 0.921). After adjustment for treatment intensity, early responders were significantly associated with complete resolution compared with non-responders (OR 2.78, 95% CI 1.03-7.45, z = 2.03, p = 0.043). Delayed responders showed a favorable but nonsignificant association (OR 1.72, 95% CI 0.69-4.31, z = 1.16, p = 0.244). Treatment intensity was positively associated with complete resolution but did not differ significantly among the response groups. Bleeding scores decreased in all groups but did not show the clear phenotype separation observed in EAT score trajectories.

Conclusions

In contrast to analyses focused on overall longitudinal changes and baseline stratification, the present study introduces a trajectory-based framework for evaluating EAT response. Treatment responses may be better understood as distinct temporal phenotypes rather than as a single uniform process. Early improvement within the first month may provide useful prognostic information, whereas delayed improvement suggests that continued treatment may still be beneficial in selected patients. These findings support evaluating EAT not only by final outcomes or overall improvement but also by the timing of improvement. As this was a single-center retrospective observational study, the results should be interpreted as hypothesis-generating rather than causal.

## Introduction

Chronic nasopharyngitis has recently been re-evaluated as a clinical entity associated not only with local symptoms, such as postnasal drip, throat discomfort, sore throat, hoarseness, and nasal obstruction, but also with a wide range of systemic symptoms, including chronic fatigue, dizziness, impaired concentration, and autonomic dysfunction [1,2]. Epipharyngeal abrasive therapy (EAT) is a treatment method developed in Japan for chronic nasopharyngitis, in which the nasopharyngeal mucosa is abraded under endoscopic observation to improve local inflammatory findings and symptoms [3]. Previous studies have reported improvements in symptoms, endoscopic findings, inflammatory findings, and bleeding findings after EAT, although substantial inter-individual variability in treatment response has also been observed [4].

Several mechanisms of action for EAT have been proposed, including suppression of inflammatory cytokines, reduction of mucosal edema, removal of inflammatory mediators through abrasion-associated bleeding and debridement, and potential modulation of systemic regulatory systems, including the autonomic nervous system [5-8]. However, these mechanisms remain partly theoretical, and empirical evidence directly explaining why individual patients improve at different time points remains limited. Therefore, describing temporal patterns of response based on clinical observational data is an important first step before discussing their possible mechanisms or clinical implications.

In clinical practice, some patients exhibit early improvement after initiation of EAT, whereas others show minimal initial response but subsequently improve with continued treatment. In contrast, some patients demonstrate little improvement despite ongoing therapy. This heterogeneity has important clinical implications for treatment continuation, timing of evaluation, patient counseling, and expectations regarding final outcomes. Nevertheless, previous studies have primarily evaluated EAT response in terms of mean longitudinal changes or final outcomes, and temporal response phenotypes, such as early responders, delayed responders, and non-responders, have not been sufficiently characterized in patients with chronic nasopharyngitis treated with EAT.

In a previous study using the same cohort of 546 patients, the author re-evaluated longitudinal changes in EAT and bleeding scores, reconstructed outcome definitions, and performed stratified analyses according to tissue type and baseline severity [9]. That study primarily focused on overall longitudinal changes and baseline-based stratification. In contrast, the present study focuses on the timing of symptom improvement during the first three months after initiation of EAT. Thus, the present analysis was designed to examine not only whether improvement occurred but also when it occurred.

Across various disease domains, treatment response is increasingly understood not as a single uniform process but as a mixture of distinct response subgroups. The presence of early responders, delayed responders, and non-responders has been reported in other clinical contexts [10-12]. However, such temporal structures of treatment response have not been sufficiently examined in the context of EAT for chronic nasopharyngitis.

The EAT three-phase adjustment theory has also suggested that clinical response to EAT may evolve over multiple temporal phases rather than following a simple dichotomous pattern of improvement versus non-improvement [13]. However, this theory remains hypothetical, and quantitative evaluation of temporal heterogeneity based on empirical clinical data remains limited.

The primary objective of this study was to classify patients with chronic nasopharyngitis treated with EAT into early responders, delayed responders, and non-responders based on changes in EAT scores during the first three months after treatment initiation. The secondary objectives were to examine the association between these temporal response phenotypes and final clinical outcomes, evaluate whether treatment intensity influenced these associations, and descriptively compare bleeding score trajectories among the response groups. Additional exploratory analyses examined whether response phenotypes differed according to chief complaint categories.

For response classification, ΔEAT was defined as the change in EAT score between two consecutive time points, calculated as ΔEAT1 = M1 − BL, ΔEAT2 = M2 − M1, and ΔEAT3 = M3 − M2, where BL indicates baseline and M1, M2, and M3 indicate months 1, 2, and 3, respectively.”

As higher EAT scores indicate greater symptom severity, negative ΔEAT values represent improvement. Through this approach, the present study aimed to introduce a trajectory-based framework for evaluating EAT response by focusing not only on whether improvement occurred but also on when improvement occurred. As this was a single-center retrospective observational study, the findings should be interpreted as exploratory and hypothesis-generating rather than causal.

## Materials and methods

Study design and participants

This study was conducted as a single-center retrospective observational study of patients with chronic nasopharyngitis treated with EAT. The study population consisted of 546 patients (100.0%) who visited the author’s institution between August 2018 and August 2019, were diagnosed with chronic nasopharyngitis, and underwent EAT.

The same cohort has been examined in the author’s previous studies. In a previous study [14], the author investigated whether clinical outcomes after EAT could be predicted from baseline characteristics using linear modeling approaches. In a subsequent study [9], outcome definitions were re-evaluated, and overall longitudinal changes in EAT and bleeding scores, as well as stratified analyses according to tissue type and baseline severity, were performed.

Although the same cohort was used, the objective of the present study differs from those of the previous studies. The previous studies focused on prediction from baseline characteristics, reconstruction of outcome definitions, overall longitudinal changes, and baseline-based stratified analyses. In contrast, the present study focused on the timing of improvement by characterizing temporal response trajectories during the first three months after EAT initiation. Therefore, the primary focus of this analysis was to describe heterogeneity in early longitudinal response patterns and evaluate their clinical implications.

In addition to the baseline characteristics of all 546 patients (100.0%), baseline characteristics were compared between the primary analysis cohort, consisting of 238 patients (43.6%) with complete EAT score data at baseline (BL), 1 month (M1), two months (M2), and three months (M3), and the excluded cohort, consisting of 308 patients (56.4%), to assess potential selection bias related to follow-up completion. The primary analysis included 238 patients (43.6%) with complete EAT score data at BL, M1, M2, and M3. This requirement was necessary because classification into the three phenotypes defined in this study - early responders, delayed responders, and non-responders - required all three indices (ΔEAT1, ΔEAT2, and ΔEAT3).

Data extraction and scoring

Clinical data were retrospectively extracted from existing medical records. Extracted variables included age, sex, disease duration, chief complaints, macroscopic tissue type, EAT scores, bleeding scores, number of EAT sessions, treatment continuation status, and final clinical outcomes. EAT scores and bleeding scores were recorded as part of routine clinical practice at the author’s institution.

As this was a single-center retrospective study based on existing medical records, a formal reliability assessment was not performed. However, EAT procedures were performed by the same operator using a consistent clinical approach, which may have reduced procedural variability within the study cohort. The absence of formal reliability assessment was considered a methodological limitation and is addressed in the "Discussion" section.

Diagnosis and outcome measures

This study included consecutive patients who visited the author’s institution with symptoms such as postnasal drip, hoarseness, foreign body sensation, headache, dizziness, and chronic fatigue and who were diagnosed with chronic nasopharyngitis based on Tanaka’s diagnostic criteria [3].

The clinical inclusion criteria were persistence of nasopharyngeal inflammatory symptoms for at least one month after onset, absence of other organic diseases in the nasopharynx, and confirmation of inflammatory findings in the nasopharynx by endoscopic examination. The clinical exclusion criteria were acute nasopharyngitis of less than one month’s duration and the presence of other diseases considered to be the primary cause of the patient’s symptoms.

Separate from these clinical exclusion criteria, patients were excluded from the primary trajectory analysis if complete EAT score data at BL, M1, M2, and M3 were unavailable. Thus, the primary trajectory analysis included 238 patients with complete longitudinal EAT score data, whereas 308 patients were excluded from the primary analysis because ΔEAT1, ΔEAT2, and ΔEAT3 could not all be calculated.

The evaluation measures included the EAT score, which represents symptom severity, and the bleeding score, which represents the degree of bleeding during abrasion [14]. The EAT score was calculated as the sum of numerical rating scale scores from 0 to 5 across 10 subjective symptom domains, with higher scores indicating greater symptom severity. The bleeding score was a graded index assessing the bleeding tendency of the nasopharyngeal mucosa during EAT, with higher scores indicating greater bleeding tendency.

Baseline variables included age, sex, disease duration, macroscopic tissue type, baseline EAT score, and baseline bleeding score. Macroscopic tissue type was classified into proliferative/hypertrophic and edematous types according to previous criteria [9]. Chief complaints were classified into 10 groups [14]. These 10 groups were further reclassified into three broader categories. Symptoms such as postnasal drip, nasal obstruction, nasal irritation, nasal dryness, abnormal sensation in the nasopharynx, sore throat, headache, shoulder stiffness, and neck stiffness were classified as nasopharyngeal local symptoms. Symptoms such as ear fullness, tinnitus, dizziness, hoarseness, and cough were classified as adjacent organ-related symptoms. Gastroesophageal reflux, dyspepsia, appetite loss, chronic fatigue, malaise, depressive symptoms, IgA nephropathy, and palmoplantar pustulosis were classified as systemic, immune-related, and autonomic-related symptoms. The association between these three categories and response phenotypes was examined.

EAT procedure and treatment frequency

EAT was performed as part of routine outpatient clinical practice at the author’s institution. All EAT procedures were performed by the same operator using a consistent technique. First, the nasopharynx was visualized by transnasal endoscopy. Transnasal EAT was then performed, followed by transoral EAT.

Treatment frequency was not determined by a fixed every-other-day protocol. In general, EAT was performed once or twice weekly, although the interval varied according to patient attendance, clinical condition, and appointment availability. To account for this real-world variation in treatment exposure, treatment intensity was quantified using monthly EAT sessions, cumulative EAT sessions through each time point, and mean treatment frequency.

Definition of response trajectories

To evaluate response trajectories during the first 0-3 months after treatment initiation, changes in EAT scores between time points were defined as follows:

ΔEAT1 = M1 − BL

ΔEAT2 = M2 − M1

ΔEAT3 = M3 − M2

As higher EAT scores indicate greater symptom severity and improvement is reflected by a decrease in score, more negative ΔEAT values indicate greater improvement.

Treatment responses were classified into three groups - early responders, delayed responders, and non-responders - based on the concept of treatment response heterogeneity and response trajectories described in recent literature. In this study, a threshold-based classification was used for clinical interpretability. Specifically, early responders were defined as patients with ΔEAT1 ≤ −6. Delayed responders were defined as those who did not meet the early responder criterion but had ΔEAT2 ≤ −4 or ΔEAT3 ≤ −4. All other patients were classified as non-responders.

**Figure 1:**
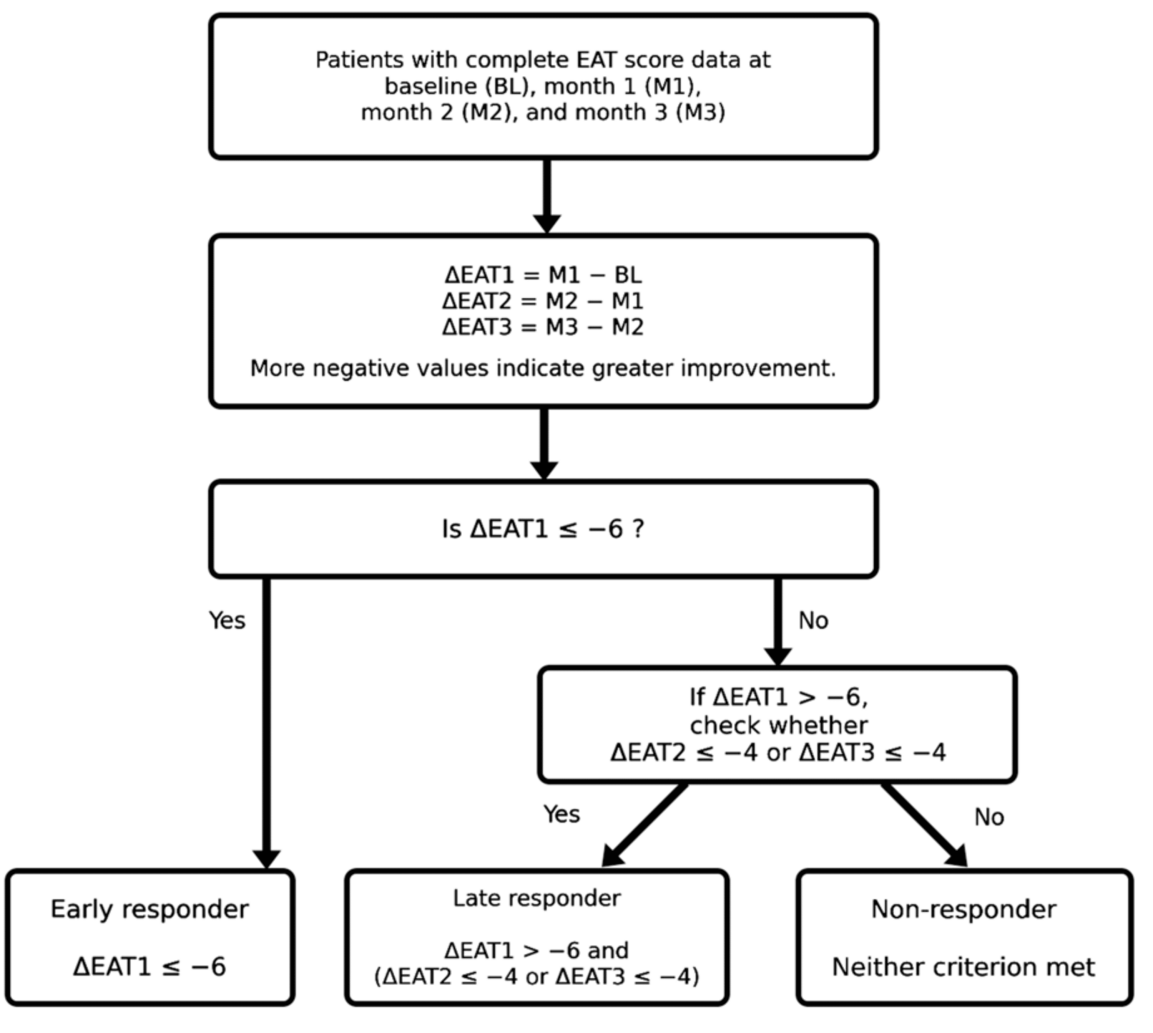
Definition of response trajectories based on ΔEAT This schematic figure illustrates response trajectory classification based on changes in EAT scores during the first three months after initiation of EAT. The primary analysis cohort included 238 patients (43.6%) with complete EAT score data at baseline (BL), one month (M1), two months (M2), and three months (M3). Changes in EAT scores were calculated as ΔEAT1 = M1 − BL, ΔEAT2 = M2 − M1, and ΔEAT3 = M3 − M2. Patients with ΔEAT1 ≤ −6 were classified as early responders. Those who did not meet the early responder criterion but had ΔEAT2 ≤ −4 or ΔEAT3 ≤ −4 were classified as delayed responders. All others were classified as non-responders. As higher EAT scores indicate greater symptom severity, negative ΔEAT values indicate improvement. No statistical test was performed for this schematic figure. BL, baseline; M1, one month; M2, two months; M3, three months; EAT, epipharyngeal abrasive therapy.

This definition was intended to identify patients with clear improvement by one month as early responders and to distinguish patients with minimal early response but subsequent improvement during months 2-3 as delayed responders. These thresholds were not based on externally established or previously validated standard criteria. Instead, they were selected as hypothesis-generating criteria to prioritize clinical interpretability, identification of clearly recognizable symptom improvement, and practical separation of response groups. Therefore, the proposed threshold-based classification should be regarded as an exploratory framework rather than a validated classification system. To reduce the influence of baseline severity and examine the robustness of this classification, a percentage-based sensitivity analysis was also performed. The definition of response trajectories is shown in Figure 1.

As a sensitivity analysis, a classification based on percentage improvement was also performed to reduce the influence of baseline severity. Percentage changes were calculated as follows:

%ΔEAT1 = (M1 − BL)/BL × 100

%ΔEAT2 = (M2 − M1)/M1 × 100

%ΔEAT3 = (M3 − M2)/M2 × 100

Early responders were defined as those with %ΔEAT1 ≤ −30%. Delayed responders were defined as those who did not meet this criterion but had %ΔEAT2 ≤ −20% or %ΔEAT3 ≤ −20%. All other patients were classified as non-responders. This percentage-based classification was used as a sensitivity analysis to assess the robustness of the threshold-based classification based on absolute score changes.

In this study, response phenotypes were defined based on changes in the EAT score, which reflects symptom changes. This approach was adopted because the primary objective of the present study was to characterize temporal response phenotypes and evaluate when patients showed symptom improvement. In contrast, the bleeding score, which reflects local mucosal findings, was not used to define response phenotypes. Instead, temporal changes in bleeding scores at BL, M1, M2, and M3 were examined descriptively and exploratorily according to response phenotype.

Definition of final outcomes and treatment intensity

Final outcomes were classified based on both EAT and bleeding score outcomes. Complete resolution was defined as cases in which both the scores met the criteria for resolution. Partial resolution was defined as cases in which either the EAT score outcome or the bleeding score outcome met the criteria for resolution. Cases in which neither the EAT score nor the bleeding score met the resolution criteria by the end of observation, or cases in which treatment was discontinued for any reason after the first month, were classified as dropouts. Cases evaluated only at baseline were classified as indeterminate.

To clearly evaluate the association between response phenotypes and final outcomes, a binary outcome was defined, with complete resolution coded as 1 and all other outcomes, including partial resolution, dropout, and indeterminate outcomes, coded as 0. Early responders, delayed responders, and non-responders, defined as symptom response trajectories to EAT, were used to evaluate their association with complete resolution.

Logistic regression models were used for the analysis. In the univariable analysis, response phenotype was included as the only explanatory variable. In the multivariable analysis, age, sex, disease duration, tissue type, baseline EAT score, and baseline bleeding score were included as covariates.

To account for the influence of treatment intensity, additional models were constructed by including either the cumulative number of EAT sessions through three months or the mean treatment frequency. Mean treatment frequency was calculated by dividing the cumulative number of EAT sessions by the corresponding observation period in weeks. Specifically, cumulative sessions were divided by 4, 8, and 12 weeks for months 1, 2, and 3, respectively. In addition, stratified sensitivity analyses were performed by dividing patients into low- and high-treatment-intensity groups based on the median cumulative number of EAT sessions through three months, and the association between response phenotype and complete resolution was evaluated within each stratum.

Statistical analysis

Statistical analyses were performed using EZR version 1.70 (Saitama Medical Center, Jichi Medical University, Saitama, Japan)​​​​​​, GraphPad Prism version 10.6.1 (GraphPad Software, Boston, MA)​​​​​​, and Python version 3.11.7 (Python Software Foundation, Wilmington, DE) with the statsmodels library version 0.14.4. All tests were two-sided, and a significance level of 5% was adopted.

First, baseline characteristics of all 546 patients, the primary analysis cohort of 238 patients, and the excluded cohort of 308 patients were summarized using descriptive statistics. Continuous variables were presented as mean ± standard deviation or median (interquartile range), depending on their distribution, and categorical variables were presented as numbers and percentages. Comparisons between the primary analysis cohort and the excluded cohort were performed using the Mann-Whitney U test for continuous variables and the χ² test for categorical variables to assess potential selection bias related to follow-up completion.

Baseline characteristics among the three response phenotypes were compared using one-way analysis of variance for continuous variables. Welch’s analysis of variance or the Kruskal-Wallis test was used when assumptions of homogeneity of variance or normality were not met. The χ² test was used for categorical variables.

Associations between response phenotypes and final outcome distribution, as well as between response phenotypes and chief complaint categories, were evaluated using the χ² test. Because some chief complaint categories contained small numbers of patients, these analyses were considered exploratory. Temporal changes in EAT scores and bleeding scores were evaluated descriptively by comparing mean values at BL, M1, M2, and M3 according to response phenotype. Figures are presented as mean values with standard errors.

The association between response phenotype and complete resolution was evaluated using logistic regression analysis. Response phenotype was modeled using dummy variables, with non-responders as the reference category. Odds ratios (ORs), 95% confidence intervals (95% CIs), Wald z statistics, and p-values were calculated. To evaluate the influence of treatment intensity, either the cumulative number of EAT sessions from zero to three months or the mean treatment frequency was entered into separate models. In addition, patients were stratified into low- and high-treatment-intensity groups based on the median cumulative number of EAT sessions, and sensitivity analyses were performed within each stratum. These analyses were conducted to account for potential confounding by treatment intensity and examine the robustness of the association between response phenotype and complete resolution.

Missing data were handled using complete-case analysis. Patients lacking EAT score data at M2 or M3 were excluded from the primary trajectory analysis because classification of delayed responders required observed values for ΔEAT2 and ΔEAT3. Multiple imputation was not performed because response phenotype classification depended on observed longitudinal EAT score changes at all four time points, and imputation of these values could have artificially assigned patients to trajectory groups. The potential selection bias introduced by complete-case analysis was assessed by comparing baseline characteristics between the primary analysis cohort and the excluded cohort and is addressed as a limitation in the "Discussion" section.

In each model, attention was paid to multicollinearity, sparse data, and instability of estimates. Results were interpreted based not only on p-values but also on ORs and 95% CIs.

Ethical considerations

This study was conducted in accordance with the Declaration of Helsinki as a retrospective observational study using existing medical records. Patient privacy was protected by anonymizing the data and restricting access. No personally identifiable information was included in the publication of results. This study was approved by the Ethics Committee of the Chiba Prefecture Health Insurance Association, with approval number 20200625006. All procedures were conducted in accordance with institutional guidelines and with respect for patient rights.

## Results

Study population

The overall study population consisted of 546 patients (100.0%). Baseline characteristics of all 546 patients (100.0%), the primary analysis cohort of 238 patients (43.6%), and the excluded cohort of 308 patients (56.4%) are shown in Table 1. The primary analysis cohort consisted of 238 patients (43.6%) with complete EAT scores at BL, M1, M2, and M3, allowing classification of response phenotypes based on ΔEAT1, ΔEAT2, and ΔEAT3.

**Table 1:** Baseline characteristics of the study population Continuous variables are presented as median (interquartile range), and categorical variables are presented as number of patients and percentage, n (%). The full cohort included 546 patients (100.0%) who underwent EAT for chronic nasopharyngitis. The primary analysis cohort included 238 patients (43.6%) with complete EAT scores at baseline (BL), one month (M1), two months (M2), and three months (M3), allowing classification of response phenotypes based on ΔEAT. The excluded cohort consisted of 308 patients (56.4%) who did not meet the complete-case criteria for the primary analysis. Comparisons were performed between the primary analysis cohort and the excluded cohort to assess potential selection bias from follow-up completion. Continuous variables were compared using the Mann–Whitney U test, and test statistics are shown as U values. Categorical variables were compared using the chi-square test with Yates’ continuity correction, and test statistics are shown as χ² values. For binary categorical variables, the same χ² statistic applies to both category rows. All tests were two-sided, and p < 0.05 was considered statistically significant. Disease duration and baseline bleeding score were significantly higher in the primary analysis cohort than in the excluded cohort. No significant differences were observed in age, sex, tissue type, or baseline EAT score. These findings suggest that the primary analysis cohort was broadly comparable to the overall cohort, although selection bias related to follow-up completion may have been present. BL, baseline; M1, 1 month; M2, 2 months; M3, 3 months; EAT, epipharyngeal abrasive therapy.

Variable	Full cohort (n = 546, 100.0%)	Primary analysis cohort (n = 238, 43.6%)	Excluded patients (n = 308, 56.4%)	Test statistic	P-value*
Age, years	50 (39–63)	51 (41–65)	47.5 (38–62)	U = 39338.5	0.142
Female, n (%)	410 (75.1)	175 (73.5)	235 (76.3)	χ² = 0.41	0.521
Male, n (%)	136 (24.9)	63 (26.5)	73 (23.7)	χ² = 0.41	0.521
Disease duration, months	8 (1–36)	12 (2–60)	6 (1–36)	U = 40894.0	0.019
Tissue type, hypertrophic/proliferative, n (%)	303 (55.5)	126 (52.9)	177 (57.5)	χ² = 0.94	0.333
Tissue type, edematous, n (%)	243 (44.5)	112 (47.1)	131 (42.5)	χ² = 0.94	0.333
Baseline EAT score	19 (13–25)	20 (14–25)	18 (13–24)	U = 39321.5	0.144
Baseline bleeding score	3 (2–3)	3 (3–3)	3 (2–3)	U = 42519.0	<0.001

Comparison of baseline characteristics between the primary analysis cohort of 238 patients (43.6%) and the excluded cohort of 308 patients (56.4%) showed no significant differences in age, sex, tissue type, or baseline EAT score. In contrast, the primary analysis cohort had a longer disease duration and higher baseline bleeding scores. These findings suggest that the primary analysis cohort was broadly similar to the overall population, although some degree of selection bias related to follow-up completion may have been present.

Classification of response types based on ΔEAT

Among the 238 patients (43.6%) included in the primary analysis, ΔEAT-based classification identified 103 patients (43.3%) as early responders, 83 patients (34.9%) as delayed responders, and 52 patients (21.8%) as non-responders. Baseline characteristics according to response phenotype using the threshold-based classification are shown in Table 2. No significant differences were observed among the three groups in age, sex, disease duration, or macroscopic tissue type.

**Table 2:** Baseline characteristics according to response type (threshold-based classification) Continuous variables are presented as mean ± standard deviation (SD) or median (interquartile range), as appropriate. Categorical variables are expressed as the number of patients and the percentage, n (%). Between-group comparisons were performed using one-way ANOVA for normally distributed continuous variables, the Kruskal–Wallis test for non-normally distributed continuous variables, and the chi-square test for categorical variables. Test statistics are reported as F values for ANOVA, H values for the Kruskal–Wallis test, and χ² values for the chi-square test. For binary categorical variables, the same χ² statistic applies to both category rows. All tests were two-sided, and p < 0.05 was considered statistically significant. Significant differences were observed in baseline EAT score (F = 18.52, p < 0.001) and baseline bleeding score (F = 5.02, p = 0.008). The baseline EAT score was highest in early responders and lowest in non-responders, suggesting that patients with greater baseline symptom burden may exhibit more pronounced reductions after treatment initiation. Similarly, baseline bleeding scores differed among groups and were lower in non-responders, suggesting that local inflammatory findings may be associated with response trajectories. No significant differences were observed in age, sex, disease duration, or tissue type. These findings suggest that response phenotypes may not be fully explained by baseline patient characteristics alone and may reflect temporal patterns of treatment response. However, as these associations are based on observational data, they should not be interpreted as causal relationships. SD, standard deviation; EAT, epipharyngeal abrasive therapy.

Variable	Early responder (n = 103, 43.3%)	Delayed responder (n = 83, 34.9%)	Non-responder (n = 52, 21.8%)	Test statistic	P-value
Age, years	49.1 ± 16.5	53.0 ± 14.9	53.0 ± 16.7	F = 1.76	0.173
Sex, male, n (%)	33 (32.0)	18 (21.7)	12 (23.1)	χ² = 2.92	0.232
Sex, female, n (%)	70 (68.0)	65 (78.3)	40 (76.9)	χ² = 2.92	
Disease duration, months	24.0 (3.0–108.0)	12.0 (3.0–36.0)	6.0 (1.0–39.0)	H = 4.22	0.121
Tissue type, hypertrophic/proliferative, n (%)	57 (55.3)	38 (45.8)	31 (59.6)	χ² = 2.87	0.238
Tissue type, edematous, n (%)	46 (44.7)	45 (54.2)	21 (40.4)	χ² = 2.87	
Baseline EAT score	23.9 ± 8.1	19.0 ± 7.3	14.6 ± 7.2	F = 18.52	<0.001
Baseline bleeding score	2.81 ± 0.47	2.81 ± 0.45	2.56 ± 0.64	F = 5.02	0.008

In contrast, baseline EAT scores differed significantly among the three groups, with the highest scores in early responders and the lowest in non-responders (one-way ANOVA, F = 27.04, p < 0.001). Baseline bleeding scores also differed significantly among groups (one-way ANOVA, F = 4.94, p = 0.008). These findings suggest that baseline symptom burden and local inflammatory findings may be associated with subsequent response trajectories.

The results of the sensitivity analysis using percentage-based classification are shown in Table 3. Among the 238 patients (43.6%), 97 (40.8%) were classified as early responders, 89 (37.4%) as delayed responders, and 52 (21.8%) as non-responders. Age differed significantly among the groups, with early responders tending to be younger than the other groups (one-way ANOVA, F = 3.03, p = 0.047). In contrast, no significant differences were observed in sex, disease duration, tissue type, baseline EAT score, or baseline bleeding score. The absence of significant baseline differences in EAT and bleeding scores suggests that percentage-based classification may reduce the influence of baseline severity to some extent. However, this classification was performed as a sensitivity analysis, and the findings derived from this observational study should not be interpreted causally. Overall, the findings of the sensitivity analysis were broadly consistent with those of the primary analysis.

**Table 3:** Baseline characteristics according to response type (percentage-based classification) Continuous variables are presented as mean ± standard deviation (SD) or median (interquartile range), as appropriate. Categorical variables are expressed as the number of patients and the percentage, n (%). Between-group comparisons were performed using one-way ANOVA for normally distributed continuous variables, the Kruskal–Wallis test for non-normally distributed continuous variables, and the chi-square test for categorical variables. Test statistics are reported as F values, H values, and χ² values, respectively. For binary categorical variables, the same χ² statistic applies to both category rows. All tests were two-sided, and p < 0.05 was considered statistically significant. A statistically significant difference was observed only for age, with early responders tending to be younger than the other groups. No significant differences were observed in sex, disease duration, tissue type, baseline EAT score, or baseline bleeding score. The lack of significant baseline differences in EAT and bleeding scores may indicate that percentage-based classification reduces the influence of baseline severity compared with threshold-based classification. However, as this was a sensitivity analysis based on observational data, the findings should not be interpreted causally. SD, standard deviation; EAT, epipharyngeal abrasive therapy.

Variable	Early responder (n = 97, 40.8%)	Delayed responder (n = 89, 37.4%)	Non-responder (n = 52, 21.8%)	Test statistic	P-value
Age, years	48.3 ± 16.2	52.9 ± 15.2	54.2 ± 16.6	F = 3.09	0.047
Sex, male, n (%)	31 (32.0)	18 (20.2)	14 (26.9)	χ² = 3.29	0.193
Sex, female, n (%)	66 (68.0)	71 (79.8)	38 (73.1)	χ² = 3.29	
Disease duration, months	12.0 (3.0–36.0)	12.0 (3.0–36.0)	12.0 (3.0–36.0)	H = 0.61	0.738
Tissue type, hypertrophic/proliferative, n (%)	58 (59.8)	39 (43.8)	29 (55.8)	χ² = 4.99	0.083
Tissue type, edematous, n (%)	39 (40.2)	50 (56.2)	23 (44.2)	χ² = 4.99	
Baseline EAT score	21.7 ± 8.2	19.0 ± 8.0	19.3 ± 9.4	F = 2.83	0.060
Baseline bleeding score	2.80 ± 0.47	2.79 ± 0.48	2.69 ± 0.58	F = 1.33	0.266

Distribution of final outcomes by response type

The distribution of final outcomes by response phenotype is shown in Table 4, and the corresponding distribution using percentage-based classification is shown in Table 5. In the threshold-based classification, no statistically significant differences were observed in the proportions of complete resolution, partial resolution, or dropout among response phenotypes (χ² test, χ² = 0.92, p = 0.921). However, descriptively, early responders and delayed responders tended to have higher proportions of complete resolution, whereas non-responders tended to have a higher proportion of dropout.

**Table 4:** Distribution of final outcomes according to response type (threshold-based classification) Final outcomes were categorized as complete resolution, partial resolution, dropout, or indeterminate. Because no patients were classified as indeterminate, this category was excluded from the chi-square analysis. Data are presented as the number of patients and the percentage, n (%). Between-group comparisons of final outcome distribution were performed using the chi-square test. As this is a multicategory contingency table, a single chi-square statistic applies to the overall outcome distribution. The test statistic is reported as the χ² value. All tests were two-sided, and p < 0.05 was considered statistically significant. No statistically significant difference was observed in the distribution of final outcomes among response types (χ² = 0.92, degrees of freedom = 4, p = 0.921). Complete resolution tended to be higher in early and delayed responders, whereas dropout tended to be higher in non-responders. As this was an univariable analysis without adjustment for baseline characteristics or treatment intensity, the findings should not be interpreted causally. EAT, epipharyngeal abrasive therapy.

Final outcome	Early responder (n = 103, 43.3%)	Delayed responder (n = 83, 34.9%)	Non-responder (n = 52, 21.8%)	Test statistic	P-value
Complete resolution	29 (28.2)	25 (30.1)	14 (26.9)	χ² = 0.92	0.921
Partial resolution	56 (54.4)	40 (48.2)	28 (53.8)		
Dropout	18 (17.5)	18 (21.7)	10 (19.2)		

**Table 5:** Distribution of final outcomes according to response type (percentage-based classification) Final outcomes were categorized as complete resolution, partial resolution, dropout, or indeterminate. Because no patients were classified as indeterminate, this category was excluded from the chi-square analysis. Data are presented as the number of patients and the percentage, n (%). Between-group comparisons were performed using the chi-square test, and a single χ² statistic applies to the overall outcome distribution. All tests were two-sided, and p < 0.05 was considered statistically significant. A significant difference in final outcome distribution was observed among response groups (χ² = 11.06, df = 4, p = 0.026). Complete resolution tended to be higher in early and delayed responders, whereas dropout was higher in non-responders. As this was an univariable analysis without adjustment for baseline characteristics or treatment intensity, the findings should not be interpreted causally. EAT, epipharyngeal abrasive therapy.

Final outcome	Early responder (n = 97, 40.8%)	Delayed responder (n = 89, 37.4%)	Non-responder (n = 52, 21.8%)	Test statistic	P-value
Complete resolution	29 (29.9)	27 (30.3)	12 (23.1)	χ² = 11.06	0.026
Partial resolution	56 (57.7)	46 (51.7)	22 (42.3)		
Dropout	12 (12.4)	16 (18.0)	18 (34.6)		

In contrast, in the percentage-based classification, non-responders showed a higher proportion of dropout and a lower proportion of complete resolution, and the distribution differed significantly among the groups (χ² test, χ² = 11.06, p = 0.026). These findings suggest that response phenotypes based on percentage improvement may more clearly reflect associations with final outcomes.

One possible explanation for this difference is that threshold-based classification may be influenced by baseline EAT scores, resulting in more severe cases being more likely to be classified as early responders. In contrast, percentage-based classification is based on relative change and may reduce the influence of baseline severity, allowing the association between response trajectories and outcomes to appear more clearly. However, this analysis was based on univariable comparisons, and these relationships should not be interpreted causally.

Temporal changes in EAT scores by response type

**Figure 2:**
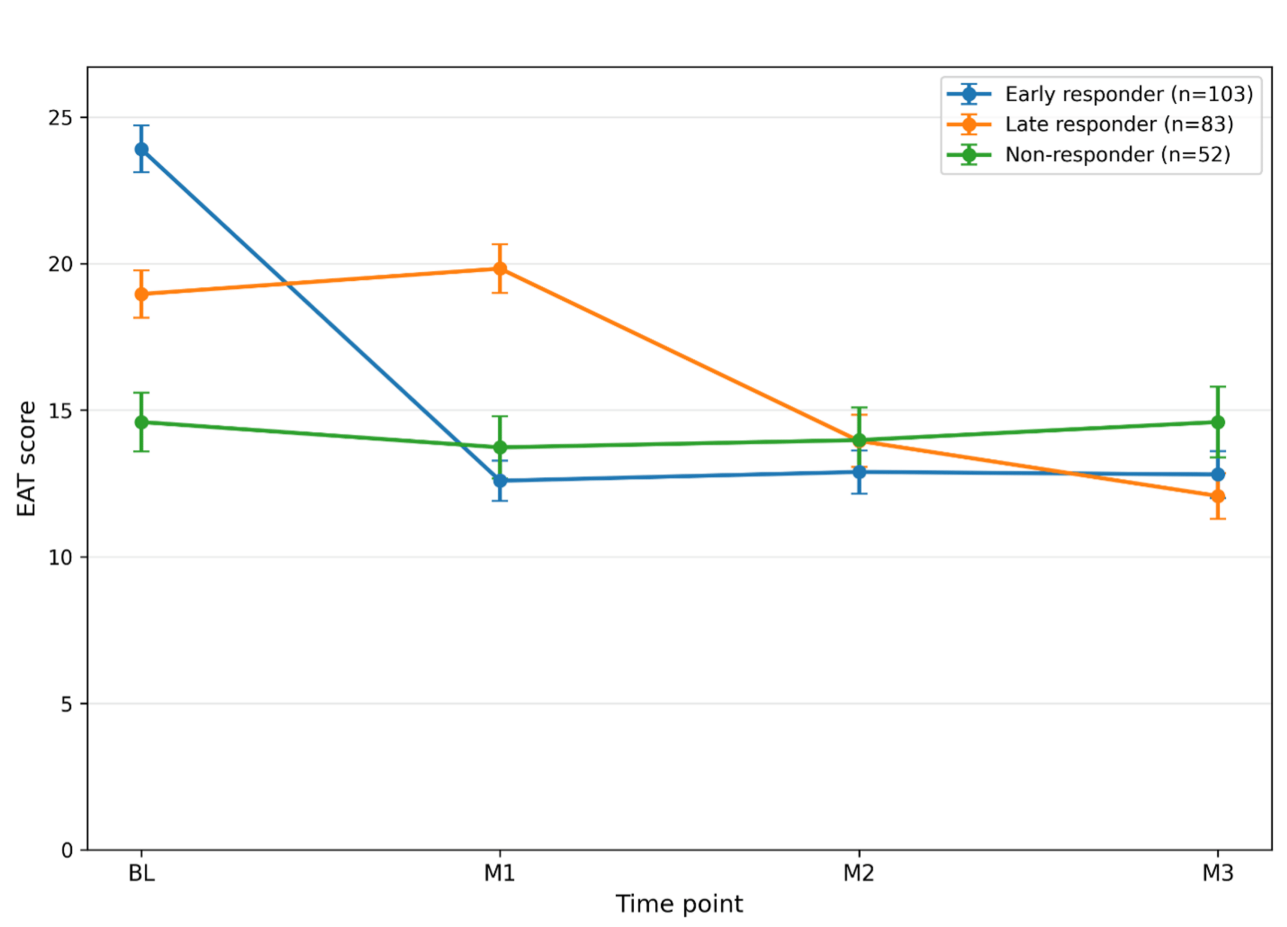
Temporal changes in EAT scores according to response type Data are presented as mean values, and error bars indicate the standard error. The findings suggest distinct temporal response trajectories among the three groups. EAT, epipharyngeal abrasive therapy.

Temporal changes in EAT scores by response phenotype are shown in Figure 2. Early responders (n = 103, 43.3%) showed a marked decrease from BL to M1, followed by a continued reduction thereafter. Delayed responders (n = 83, 34.9%) showed a relatively modest reduction at M1, with clearer decreases at M2 and M3. Non-responders (n = 52, 21.8%) showed only small reductions overall and demonstrated less improvement than the other two groups. Thus, temporal changes in EAT scores were visually distinguishable as three temporal phenotypes: early responders, delayed responders, and non-responders.

Temporal changes in bleeding scores by response type

**Figure 3:**
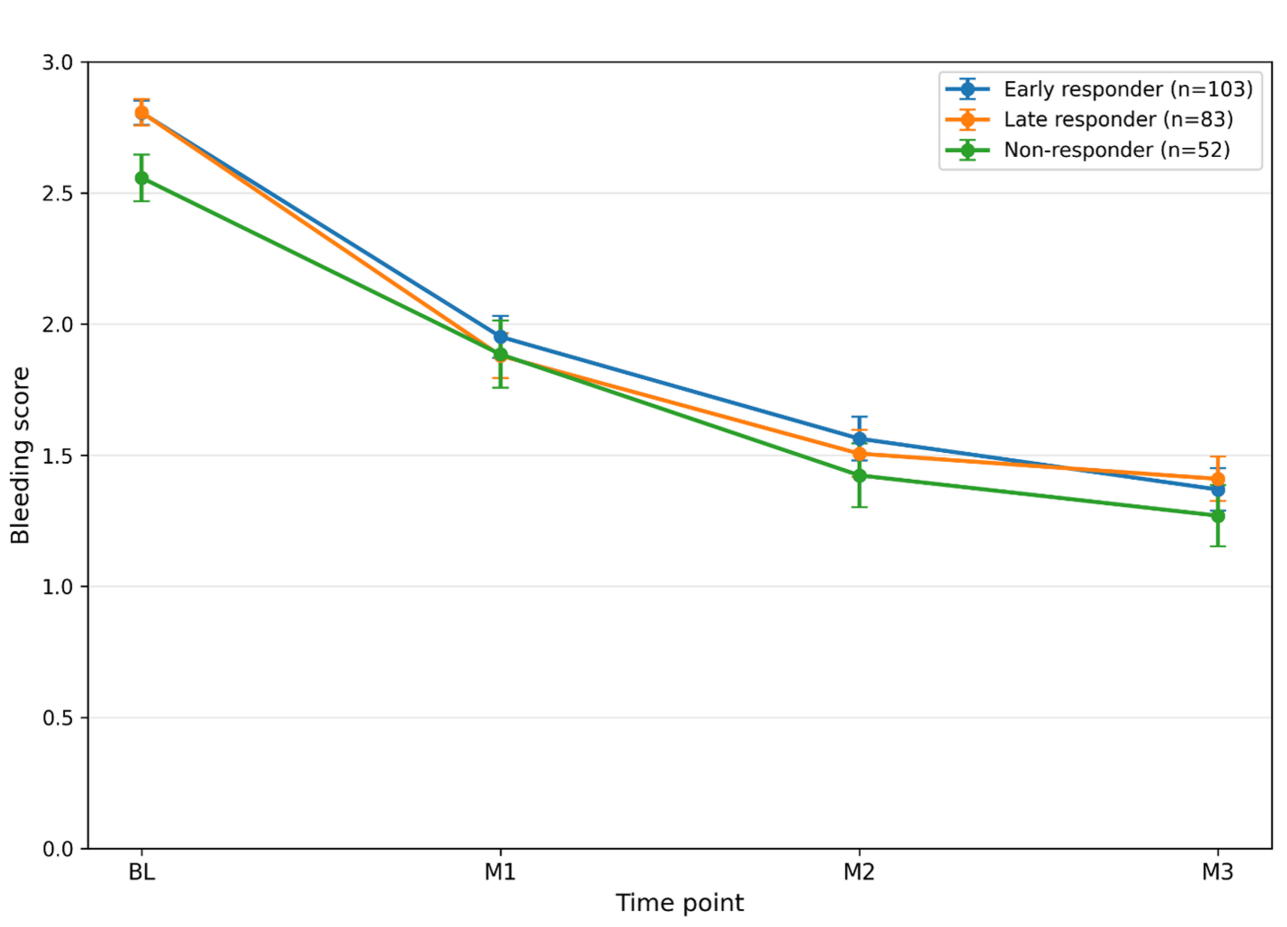
Temporal changes in bleeding scores according to response type Data are presented as mean values, and error bars indicate the standard error. This figure is descriptive, and no statistical comparison was performed. Bleeding scores decreased over time in all three groups and exhibited similar patterns of improvement across response types. In contrast to the clear separation observed in EAT scores (Figure 2), no distinct between-group differences were observed in bleeding score trajectories. The temporal changes in bleeding scores appeared relatively homogeneous across the groups. These findings suggest that response classification based on ΔEAT primarily reflects temporal characteristics of symptom changes rather than local bleeding findings, and that the trajectories of bleeding score improvement may follow a different, more uniform course.

Temporal changes in bleeding scores by response phenotype are shown in Figure 3. Mean bleeding scores decreased over time from baseline to three months in all three groups. Baseline bleeding scores were 2.81 in early responders (n = 103, 43.3%), 2.81 in delayed responders (n = 83, 34.9%), and 2.56 in non-responders (n = 52, 21.8%), decreasing to 1.37, 1.41, and 1.27 at three months, respectively.

In contrast to EAT scores, the trajectories of bleeding scores were broadly similar across the three groups, and no clear separation among the three phenotypes was observed. These findings suggest that response phenotypes defined by EAT scores were not clearly reflected in the temporal changes in local bleeding findings.

Treatment intensity indicators by response type

Treatment intensity indicators by response phenotype are shown in Table 6. The cumulative number of EAT sessions at one, two, and three months did not differ significantly among early responders (n = 103, 43.3%), delayed responders (n = 83, 34.9%), and non-responders (n = 52, 21.8%). The cumulative number of EAT sessions through three months also did not differ significantly among the three groups (one-way ANOVA, F = 0.18, p = 0.835). Similarly, no significant differences were observed in mean treatment frequency or monthly treatment counts. These findings suggest that differences in temporal improvement patterns among response phenotypes were not sufficiently explained by treatment-intensity indicators alone.

**Table 6:** Treatment intensity indicators according to response type Continuous variables are presented as mean ± standard deviation (SD). Between-group comparisons were performed using one-way analysis of variance (ANOVA). Test statistics are reported as F values. All tests were two-sided, and p < 0.05 was considered statistically significant. Mean treatment frequency was calculated by dividing the cumulative EAT sessions by 4, 8, and 12 weeks for months 1, 2, and 3, respectively. No statistically significant differences were observed among the three response groups for monthly EAT sessions, cumulative EAT sessions, or mean treatment frequency at any time point. These findings suggest that treatment intensity alone did not explain differences in response trajectories. SD, standard deviation; EAT, epipharyngeal abrasive therapy.

Variable	Early responders (n = 103, 43.3%)	Delayed responders (n = 83, 34.9%)	Non-responders (n = 52, 21.8%)	Test statistic	P-value
EAT sessions (Month 1)	4.94 ± 1.71	4.73 ± 1.94	5.21 ± 3.04	F = 0.81	0.453
EAT sessions (Month 2)	4.31 ± 2.15	4.43 ± 2.40	4.60 ± 2.58	F = 0.27	0.770
EAT sessions (Month 3)	4.07 ± 2.62	3.99 ± 2.35	3.94 ± 1.89	F = 0.06	0.947
Cumulative EAT sessions (Month 1)	4.94 ± 1.71	4.73 ± 1.94	5.21 ± 3.04	F = 0.81	0.453
Cumulative EAT sessions (Month 2)	9.25 ± 3.29	9.17 ± 3.77	9.81 ± 5.36	F = 0.46	0.632
Cumulative EAT sessions (Month 3)	13.32 ± 5.29	13.16 ± 5.59	13.75 ± 6.39	F = 0.18	0.835
Mean treatment frequency (Month 1)	1.24 ± 0.43	1.18 ± 0.48	1.30 ± 0.76	F = 0.81	0.453
Mean treatment frequency (Month 2)	1.16 ± 0.41	1.15 ± 0.47	1.23 ± 0.67	F = 0.46	0.632
Mean treatment frequency (Month 3)	1.11 ± 0.44	1.10 ± 0.47	1.15 ± 0.53	F = 0.18	0.835

Factors associated with complete resolution

To examine factors associated with complete resolution, multivariable logistic regression analyses including treatment intensity were performed. The results are shown in Table 7. In Model 1, the cumulative number of EAT sessions over three months was included as the treatment-intensity indicator. In Model 2, mean treatment frequency over three months was included as the treatment-intensity indicator.

**Table 7:** Multivariable logistic regression analysis for complete resolution Logistic regression analysis was performed with complete resolution as the dependent variable. Complete resolution was coded as 1, and all other outcomes were coded as 0. Results are presented as ORs with 95% CIs, Wald z statistics, and p-values. Model 1 was adjusted for cumulative EAT sessions from 0 to 3 months, whereas Model 2 was adjusted for mean treatment frequency over the same period. Both models also included response phenotype, age, sex, disease duration, tissue type, baseline EAT score, and baseline bleeding score as covariates, with non-responders as the reference category. P-values were calculated using Wald tests, and the corresponding test statistics are reported as Wald z values. All tests were two-sided, and p < 0.05 was considered statistically significant. Early responders were significantly associated with complete resolution compared with non-responders in both models. Delayed responders did not show a statistically significant association, although the ORs consistently indicated a favorable direction. Age was positively associated with complete resolution, whereas baseline EAT score was negatively associated with complete resolution. Sex, disease duration, tissue type, and baseline bleeding score were not significantly associated with complete resolution. Treatment intensity indicators were included as adjustment factors in each model. The association between response phenotype and complete resolution may persist after accounting for treatment intensity. However, because this analysis was based on observational data, the findings should not be interpreted as indicating a causal relationship. OR, odds ratio; CI, confidence interval; EAT, epipharyngeal abrasive therapy.

Variable	Model 1: Adjusted for cumulative EAT sessions OR (95% CI)	Wald z	P-value	Model 2: Adjusted for mean treatment frequency OR (95% CI)	Wald z	P-value
Early vs non-responder	2.78 (1.03–7.45)	2.03	0.043	2.78 (1.03–7.45)	2.03	0.043
Delayed vs non-responder	1.72 (0.69–4.31)	1.16	0.244	1.72 (0.69–4.31)	1.16	0.244
Age, per one-year increase	1.03 (1.01–1.05)	2.57	0.010	1.03 (1.01–1.05)	2.57	0.010
Sex, female vs male	1.64 (0.72–3.74)	1.19	0.236	1.64 (0.72–3.74)	1.19	0.236
Disease duration, per one-month increase	1.00 (1.00–1.01)	1.18	0.238	1.00 (1.00–1.01)	1.18	0.238
Tissue type, edematous vs hypertrophic/proliferative	1.61 (0.81–3.22)	1.35	0.177	1.61 (0.81–3.22)	1.35	0.177
Baseline EAT score, per one-point increase	0.93 (0.89–0.97)	−3.09	0.002	0.93 (0.89–0.97)	−3.09	0.002
Baseline bleeding score, per one-point increase	0.93 (0.50–1.71)	−0.25	0.804	0.93 (0.50–1.71)	−0.25	0.804
Cumulative EAT sessions, per one-session increase	1.08 (1.01–1.15)	2.41	0.016	—	—	—
Mean treatment frequency, per one session/month increase	—	—	—	1.25 (1.04–1.50)	2.41	0.016

In both models, early responders were significantly associated with complete resolution compared with non-responders (Model 1 and Model 2: OR 2.78, 95% CI 1.03-7.45, z = 2.03, p = 0.043). In contrast, delayed responders did not show a statistically significant association (OR 1.72, 95% CI 0.69-4.31, z = 1.16, p = 0.244).

Among covariates, age was positively associated with complete resolution (OR 1.03 per one-year increase, 95% CI 1.01-1.05, p = 0.010), whereas baseline EAT score was negatively associated with complete resolution (OR 0.93 per one-point increase, 95% CI 0.89-0.97, p = 0.002). No significant associations were observed for sex, disease duration, tissue type, or baseline bleeding score.

Regarding treatment intensity indicators, both cumulative EAT sessions and mean treatment frequency were positively associated with complete resolution (both p = 0.016). These findings suggest that the association between early response and complete resolution may persist after accounting for treatment intensity.

Stratified sensitivity analysis by treatment intensity

To further examine the influence of treatment intensity, patients were stratified into a low-treatment-intensity group of 124 patients (52.1%) and a high-treatment-intensity group of 114 patients (47.9%) based on the median cumulative number of EAT sessions through three months, which was 12 sessions. The association between response phenotype and complete resolution was then evaluated within each stratum (Table 8).

**Table 8:** Stratified sensitivity analysis by treatment intensity A stratified sensitivity analysis was performed to evaluate the association between ΔEAT-based response phenotypes and complete resolution according to treatment intensity. Patients were stratified into two groups based on the median cumulative number of EAT sessions through month 3 (12 sessions): low-treatment intensity (≤12 sessions, n = 124) and high-treatment intensity (≥13 sessions, n = 114). Within each stratum, logistic regression models were used to estimate ORs and 95% CIs for complete resolution. Early responders and delayed responders were compared with non-responders as the reference group. Test statistics are reported as Wald z values, and p-values were calculated using Wald tests. All tests were two-sided, and p < 0.05 was considered statistically significant. In both strata, early responders showed higher ORs for complete resolution compared with non-responders, indicating a consistent direction of association across treatment-intensity levels. Statistical significance was observed in the high-treatment intensity group but not in the low-treatment intensity group; however, CIs overlapped between strata. These findings suggest that the association between early response and complete resolution may not be fully explained by treatment intensity alone. However, because this analysis was based on stratification with a reduced sample size, the estimates are less precise and should be interpreted as exploratory. OR, odds ratio; CI, confidence interval; EAT, epipharyngeal abrasive therapy.

Variable	Low-treatment intensity (≤12 sessions, n = 124) OR (95% CI)	Wald z	P-value	High-treatment intensity (≥13 sessions, n = 114) OR (95% CI)	Wald z	P-value
Early vs non-responder	2.65 (0.95–7.37)	1.86	0.062	2.81 (1.05–7.53)	2.04	0.041
Delayed vs non-responder	1.50 (0.58–3.87)	0.84	0.400	1.80 (0.70–4.60)	1.23	0.220

In interpreting this analysis, it is important to distinguish between two separate questions: whether patients who show early response are more likely to achieve complete resolution and whether treatment intensity determines the occurrence of early response. The former evaluates how the state of early response is associated with subsequent outcomes and represents the primary focus of this analysis. The latter addresses a different analytical question regarding whether treatment intensity determines the development of early response.

As shown in Table 6, no significant differences in treatment intensity were observed among the three response phenotypes in this study. Therefore, within the scope of the present analysis, the occurrence of early response could not be explained by treatment intensity alone.

In the stratified analysis, early responders showed higher ORs for complete resolution than non-responders in both the low- and high-treatment-intensity groups, and the direction of association was consistent. Statistical significance was observed in the high-treatment-intensity group but not in the low-treatment-intensity group; however, CIs were wide. Therefore, statistical significance alone should not be interpreted as evidence of a difference in effect between the two strata.

These findings suggest that the association between early response and complete resolution may not be sufficiently explained by treatment intensity differences alone. However, this analysis did not directly examine whether treatment intensity causes early response; therefore, no conclusion can be drawn from this study regarding whether treatment intensity determines the development of early response.

Association with chief complaint categories

The association between the 10 chief complaint categories and response phenotypes is shown in Table 9. No significant association was observed between the 10 chief complaint categories and response phenotypes (χ² test, χ² = 18.30, p = 0.436). However, some categories contained a very small number of patients, limiting the stability of the estimates.

**Table 9:** Association between response type and 10 categories of chief complaints The association between the 10 categories of chief complaints and response phenotypes based on ΔEAT was examined. Patients were classified according to their chief complaints, and the distribution of response phenotypes within each chief complaint category was compared. Data are presented as the number of patients and the percentage, n (%). Percentages in the response phenotype columns represent row percentages within each chief complaint category. Percentages in the total column represent the proportion of each chief complaint category among the primary analysis cohort. Between-group comparisons were performed using the chi-square test. As this is a multicategory contingency table, a single chi-square statistic applies to the overall distribution across all chief complaint categories. The test statistic is reported as the χ² value. All tests were two-sided, and p < 0.05 was considered statistically significant. No statistically significant association was observed between the 10 chief complaint categories and response phenotypes (χ² = 18.30, degrees of freedom = 18, p = 0.436). However, several categories contained very small numbers of patients, which may limit the estimate stability and chi-square interpretability. These findings suggest that chief complaints at the initial visit alone may not sufficiently explain differences in response trajectories following EAT. However, as this analysis was based on univariable observational data, the findings should not be interpreted as indicating a causal relationship. EAT, epipharyngeal abrasive therapy.

Chief complaint category	Early responder, n (%)	Delayed responder, n (%)	Non-responder, n (%)	Total, n (%)	Test statistic	P-value
1. Postnasal drip-related symptoms	45 (44.6)	40 (39.6)	16 (15.8)	101 (42.4)	χ² = 18.30	0.436
2. Nasopharyngeal irritation-related symptoms	17 (36.2)	13 (27.7)	17 (36.2)	47 (19.7)		
3. Nasal-related symptoms	13 (50.0)	10 (38.5)	3 (11.5)	26 (10.9)		
4. Eustachian tube-related symptoms	4 (36.4)	4 (36.4)	3 (27.3)	11 (4.6)		
5. Dizziness/facial nerve-related symptoms	1 (33.3)	1 (33.3)	1 (33.3)	3 (1.3)		
6. Voice-related symptoms	8 (53.3)	4 (26.7)	3 (20.0)	15 (6.3)		
7. Laryngopharyngeal irritation-related symptoms	11 (47.8)	8 (34.8)	4 (17.4)	23 (9.7)		
8. Sleep apnea-related symptoms	0 (0.0)	1 (100.0)	0 (0.0)	1 (0.4)		
9. Gastrointestinal/allergy/immune/systemic symptoms	1 (20.0)	1 (20.0)	3 (60.0)	5 (2.1)		
10. Autonomic-related symptoms	3 (50.0)	1 (16.7)	2 (33.3)	6 (2.5)		

The association between the three broader chief complaint categories and response phenotypes is shown in Table 10. Among patients with nasopharyngeal local symptoms (n = 174, 73.1%), 75 (43.1%) were early responders, 63 (36.2%) were delayed responders, and 36 (20.7%) were non-responders. Among those with adjacent organ-related symptoms (n = 52, 21.8%), the corresponding numbers were 24 (46.2%), 17 (32.7%), and 11 (21.2%), respectively. In the systemic/immune/autonomic-related symptom group (n = 12, 5.0%), 4 patients (33.3%) were early responders, 3 (25.0%) were delayed responders, and 5 (41.7%) were non-responders. No significant association was observed between the three chief complaint categories and response phenotypes (χ² = 3.15, p = 0.534).

**Table 10:** Association between three major chief complaint categories and response phenotypes The 10 chief complaint categories were reclassified into three broader groups: nasopharyngeal local symptoms, adjacent organ-related symptoms, and systemic/immune/autonomic symptoms. The association between these three chief complaint categories and response phenotypes based on ΔEAT was examined. Data are presented as the number of patients and the percentage, n (%). Percentages in the response phenotype columns represent row percentages within each chief complaint category. Percentages in the total row represent the distribution of response phenotypes in the primary analysis cohort. Percentages in the total column represent the proportion of each chief complaint category among the primary analysis cohort. Between-group comparisons were performed using the chi-square test. As this is a multicategory contingency table, a single chi-square statistic applies to the overall distribution across all chief complaint categories. The test statistic is reported as the χ² value. All tests were two-sided, and p < 0.05 was considered statistically significant. No statistically significant association was observed between the three major chief complaint categories and response phenotypes (χ² = 3.15, degrees of freedom = 4, p = 0.534). These findings suggest that broad chief complaint categories alone may not sufficiently explain response trajectory differences following EAT. However, as this analysis was based on univariable observational data, the findings should not be interpreted as indicating a causal relationship. EAT, epipharyngeal abrasive therapy.

Chief complaint category	Early responder, n (%)	Delayed responder, n (%)	Non-responder, n (%)	Total, n (%)	Test statistic	P-value
Nasopharyngeal local symptoms	75 (43.1)	63 (36.2)	36 (20.7)	174 (73.1)	χ² = 3.15	0.534
Adjacent organ-related symptoms	24 (46.2)	17 (32.7)	11 (21.2)	52 (21.8)		
Systemic/immune/autonomic symptoms	4 (33.3)	3 (25.0)	5 (41.7)	12 (5.0)		
Total	103 (43.3)	83 (34.9)	52 (21.8)	238 (100.0)		

## Discussion

Principal findings

In this study, treatment responses after EAT for chronic nasopharyngitis were classified into three temporal phenotypes - early responders, delayed responders, and non-responders - based on changes in EAT scores during the first three months of treatment. Among 238 patients with complete longitudinal EAT score data from baseline to three months, 103 patients (43.3%) were classified as early responders, 83 patients (34.9%) as delayed responders, and 52 patients (21.8%) as non-responders.

The EAT score trajectories differed visually and clinically among these response phenotypes. Early responders showed marked improvement within the first month; delayed responders showed later improvement during months 2-3; and non-responders showed limited improvement during the observation period. In the threshold-based classification, the distribution of final outcomes did not differ significantly among response groups. However, in multivariable logistic regression models adjusted for baseline characteristics and treatment intensity, early responders were significantly associated with complete resolution compared with non-responders. Delayed responders showed ORs in a favorable direction, although this association did not reach statistical significance.

These findings suggest that treatment response to EAT is a heterogeneous temporal process rather than a uniform pattern of improvement. However, because this study was retrospective and observational, these response phenotypes should be interpreted as clinically observed temporal categories and not as causal or biologically established subtypes.

Temporal response phenotypes and clinical interpretation

The main contribution of this study is the introduction of a trajectory-based framework for evaluating EAT response. Previous analyses using the same cohort primarily examined baseline predictors, reconstructed outcome definitions, overall longitudinal changes, and stratified analyses according to baseline severity or tissue type. In contrast, the present study focused on the timing of symptom improvement during the early treatment period.

This distinction is clinically relevant because patients who do not improve immediately after EAT initiation are not a homogeneous group. Some patients may show delayed improvement after continued treatment, whereas others may show limited improvement throughout the first three months. Evaluating only baseline severity or final outcome may therefore overlook clinically meaningful differences in the timing of response.

At the same time, the proposed phenotypes should be interpreted cautiously. The classification was based on exploratory ΔEAT thresholds selected for clinical interpretability and practical group separation, not on externally validated criteria. The early responder, delayed responder, and non-responder categories should be regarded as a hypothesis-generating framework requiring external validation rather than an established classification system.

Baseline severity, response phenotype, and final outcome

Baseline EAT scores were highest in early responders and lowest in non-responders in the threshold-based classification. Baseline bleeding scores also differed among groups and were lower in non-responders. These findings suggest that patients with greater baseline symptom burden or more apparent local inflammatory findings may show larger absolute score reductions after treatment initiation.

However, multivariable analysis showed that baseline EAT score was negatively associated with complete resolution. This indicates that a large early reduction in symptom score and achievement of final complete resolution are not identical clinical concepts. Patients with higher baseline symptom severity may have more room for score reduction but remain less likely to meet strict criteria for complete resolution. Therefore, response phenotype and final outcome should be interpreted as related but distinct dimensions of treatment evaluation.

EAT score trajectories and bleeding score trajectories

A notable finding was that the three response phenotypes were more clearly separated by EAT score trajectories than by bleeding score trajectories. Bleeding scores decreased over time in all three groups, but the patterns were broadly similar among early responders, delayed responders, and non-responders.

This suggests that the ΔEAT-based classification primarily captures the temporal pattern of symptom response rather than that of local bleeding findings. Symptom improvement and local mucosal bleeding improvement may not necessarily occur along the same time axis. This is important because EAT scores and bleeding scores may reflect different aspects of chronic nasopharyngitis and its response to treatment.

Ohno reported that bleeding findings may be useful for assessing pretreatment severity, whereas caution is needed when using bleeding as an outcome measure [15]. Consistent with this view, the present findings suggest that symptom scores and local bleeding findings should not be treated as interchangeable indicators of EAT response. However, because this study did not directly examine the biological basis of bleeding changes, the reason for the divergence between these trajectories remains uncertain.

Treatment intensity and response phenotypes

Treatment intensity should be considered when interpreting response trajectories. In this study, no significant differences were observed among the three response phenotypes in monthly EAT sessions, cumulative EAT sessions, or mean treatment frequency, suggesting that, within the primary analysis cohort, differences in temporal response patterns were not sufficiently explained by the treatment volume alone.

In contrast, when complete resolution was the dependent variable, both cumulative EAT sessions and mean treatment frequency were positively associated with outcome. This indicates that treatment intensity may be related to final outcome, even if it does not fully explain the timing of symptom improvement. Temporal response phenotype and cumulative treatment exposure may thus provide overlapping but distinct information.

In stratified sensitivity analyses according to treatment intensity, early responders showed higher odds ratios for complete resolution than non-responders in both low- and high-treatment-intensity strata, and the direction of association was generally consistent. However, stratification reduced the sample size, and the CIs were wide; so, these findings should be interpreted as exploratory. They do not establish that early response is independent of treatment intensity in a causal sense but suggest that the observed association between early response and complete resolution was not fully explained by simple treatment-count indicators alone.

Threshold-based and percentage-based classifications

The threshold-based ΔEAT classification and the percentage-based classification provided complementary information. In the threshold-based classification, final outcome distribution did not differ significantly among response groups, although early and delayed responders tended to have higher proportions of complete resolution, and non-responders tended to have a higher proportion of dropout. In contrast, the percentage-based classification showed a significant difference in final outcome distribution, with non-responders showing a higher proportion of dropout and a lower proportion of complete resolution.

One possible explanation is that threshold-based classification using absolute score changes is influenced by baseline EAT score. Patients with higher baseline symptom severity may be more likely to meet an absolute reduction threshold, even if they do not ultimately achieve complete resolution. Percentage-based classification may partially reduce the influence of baseline severity by evaluating relative change. Therefore, this sensitivity analysis suggests that the method used to define response phenotypes can influence the apparent association between response trajectories and outcomes.

These findings should not, however, be interpreted as demonstrating that percentage-based classification is superior to threshold-based classification. The percentage-based analysis was exploratory and was not fully adjusted for baseline characteristics or treatment intensity in the same way as the multivariable models. Rather, the results suggest that different operational definitions of response phenotype may capture different aspects of treatment response, and that future studies should compare and validate these definitions prospectively.

Interpretation of delayed responders

The delayed responder group requires careful interpretation. Delayed responders did not show a statistically significant independent association with complete resolution compared with non-responders. However, their odds ratios were consistently favorable across analyses.

This finding suggests that a lack of marked improvement within the first month should not automatically be interpreted as treatment failure. Some patients may show delayed improvement during months 2-3. Clinically, this supports the importance of evaluating EAT response over a sufficient time window rather than relying only on very early symptom changes. Nevertheless, because the estimates for delayed responders were imprecise and did not reach statistical significance, this interpretation should be considered exploratory. The clinical usefulness of identifying delayed responders requires confirmation in larger prospective cohorts.

Mechanistic interpretation and the EAT three-phase adjustment theory

The observed temporal phenotypes appear conceptually compatible with the EAT three-phase adjustment theory, which proposes that responses to EAT may occur across multiple temporal phases rather than through a single uniform process [13]. Early responders, delayed responders, and non-responders may be interpreted as clinical patterns that are broadly consistent with this conceptual framework.

However, this study does not validate the EAT three-phase adjustment theory. The theory remains hypothetical, and the present study did not directly measure inflammatory cytokines, autonomic nervous system function, histological changes, immune cell dynamics, or other biological markers. Therefore, the observed response phenotypes cannot be directly linked to specific pathophysiological mechanisms.

Similarly, previous reports have suggested possible anti-inflammatory or immunological effects of EAT. For example, Mogitate reported improvement in symptoms and local findings after EAT, along with changes in inflammation-related findings and a decrease in CD4-positive T cells [16]. These findings provide a biological rationale for further investigation. However, the present study did not measure such biological changes. Therefore, it cannot be concluded that early responders represent a specific immunological subtype or that delayed responders reflect a specific neuroregulatory mechanism. The three phenotypes identified in this study should be understood as clinically observed temporal categories, and their biological basis remains a subject for future research.

Clinical implications

The present findings suggest that EAT response may be evaluated not only by whether improvement occurs or whether complete resolution is eventually achieved but also by the timing of symptom improvement. Early response may provide useful prognostic information regarding the subsequent clinical course. Delayed response may indicate that some patients with limited early improvement can still improve with continued observation over a two- to three-month period.

These clinical implications should be interpreted conservatively. The proposed trajectory-based framework has not been externally validated and should not be used as a definitive clinical decision-making tool. The findings do not directly indicate when treatment should be continued, modified, or discontinued. Instead, they suggest that temporal response patterns may provide additional information for treatment monitoring, patient counseling, and the design of future prospective studies.

In patients with little symptom improvement through three months, re-evaluation of comorbid conditions, reconsideration of treatment goals, or use of additional assessment methods may be clinically reasonable. However, such decisions should be made individually and cannot be determined from this observational study alone.

Limitations

This study has several important limitations.

First, this was a single-center retrospective observational study. Patient characteristics, EAT technique, treatment frequency, follow-up decisions, and outcome assessment were all influenced by the clinical practice of a single institution. Although the use of a single operator and a consistent clinical approach may have reduced procedural variability within the study cohort, it may also limit generalizability to other institutions where the EAT technique, treatment intervals, and follow-up policies differ.

Second, this study did not include a control group. Therefore, it cannot determine whether the observed changes in EAT scores, the identified response trajectories, or the associations with final outcomes were specific effects of EAT itself. Natural disease course, regression to the mean, concomitant treatments, healthcare-seeking behavior, patient motivation, or the passage of time may have contributed to the observed findings. Accordingly, the results should be interpreted as associations observed during EAT treatment, not as evidence of causal treatment effects.

Third, the primary analysis was based on complete-case analysis restricted to 238 patients (43.6%) with complete EAT score data from baseline to three months. This was necessary because the classification of delayed responders required observed values at M2 and M3. However, excluding 308 of 546 patients raises concern for selection bias. Although the primary analysis cohort and excluded cohort did not differ significantly in age, sex, tissue type, or baseline EAT score, the primary analysis cohort had longer disease duration and higher baseline bleeding scores. Therefore, the findings may be more applicable to patients who continued follow-up and may not fully generalize to the original cohort or all patients treated with EAT.

Fourth, the response phenotype thresholds were not externally validated. The ΔEAT cutoffs used to define early and delayed responders were selected for clinical interpretability and practical group separation, not derived from validated statistical or biological criteria. Therefore, the classification should be regarded as exploratory and hypothesis-generating. Future studies should validate whether the same thresholds, or alternative data-driven thresholds, produce reproducible findings in independent cohorts.

Fifth, missing data may have influenced the results. Delayed responder classification is particularly sensitive to missing data because it requires EAT score information at later time points. Multiple imputation was not performed because assigning imputed EAT scores could have artificially assigned patients to trajectory groups. However, complete-case analysis may introduce bias if missingness was related to symptoms, treatment response, treatment burden, or patient motivation.

Sixth, residual confounding remains possible. Although the multivariable models adjusted for age, sex, disease duration, tissue type, baseline EAT score, baseline bleeding score, and treatment intensity, unmeasured confounders such as comorbidities, concomitant medications, lifestyle factors, healthcare-seeking behavior, and clinician decisions regarding treatment continuation were not fully captured. Treatment intensity itself may reflect both clinical need and the patient’s ability or willingness to continue visits. Therefore, associations adjusted for treatment intensity should not be interpreted as causal.

Seventh, several analyses were exploratory. Subgroup analyses and stratified sensitivity analyses reduced the sample size and produced wide CIs. Formal adjustment for multiple comparisons was not performed, which increases the possibility of type I error. In addition, the number of complete resolution events was limited relative to the number of covariates in some logistic regression models, and model estimates may therefore be unstable. These statistical limitations require cautious interpretation of the regression results.

Eighth, the final outcome definition was reconstructed from the author’s previous work using the same cohort. Although this definition was intended to distinguish complete resolution from partial resolution and dropout, different outcome definitions may yield different distributions and associations. Therefore, the observed relationship between response phenotype and final outcome may depend partly on the outcome framework used in this study.

Ninth, the bleeding score was a semiquantitative ordinal measure and may have been less sensitive than symptom scores for detecting subtle differences among response phenotypes. In addition, a formal reliability assessment of scoring was not performed because this was a retrospective study based on existing clinical records. Therefore, measurement variability cannot be fully excluded.

Tenth, this study did not directly measure biological mechanisms. Inflammatory markers, autonomic function, immune parameters, histological findings, and other mechanistic indicators were not assessed. As a result, the response phenotypes identified in this study cannot be interpreted as definitive biological subtypes. Any discussion of inflammatory, autonomic, immunological, or EAT 3-phase adjustment theory-related mechanisms should be regarded as speculative and hypothesis-generating.

Finally, the analysis of chief complaint categories was exploratory and limited by small numbers in some categories, overlapping symptoms, and possible classification errors. The absence of a significant association between chief complaint category and response phenotype does not prove that symptom domains are unrelated to treatment response. Rather, it suggests only that, within the present classification framework, chief complaint categories alone did not explain temporal response trajectories.

Future studies should prospectively evaluate larger and more diverse cohorts, apply standardized EAT protocols and outcome assessments, validate response phenotype definitions externally, and incorporate biological markers to clarify whether the observed temporal phenotypes correspond to specific pathophysiological processes.

## Conclusions

In this single-center retrospective observational study, treatment responses to EAT for chronic nasopharyngitis showed heterogeneous temporal patterns during the first three months of treatment. Based on changes in EAT scores, patients were classified into early responders, delayed responders, and non-responders. EAT response may therefore be better understood as a temporal process rather than a uniform pattern of improvement. Early response was associated with complete resolution after adjustment for baseline characteristics and treatment intensity, suggesting that symptom improvement within the first month may provide useful prognostic information. Delayed responders showed a favorable but statistically nonsignificant trend compared with non-responders, indicating that lack of marked improvement during the first month should not necessarily be interpreted as treatment failure.

Treatment intensity, chief complaint categories, and bleeding score trajectories alone did not fully explain the observed differences in symptom response trajectories. The discrepancy between EAT score and bleeding score trajectories suggests that symptom improvement and local bleeding findings may reflect different aspects of treatment response. These findings should, however, be interpreted cautiously. The response phenotypes were based on exploratory thresholds that have not been externally validated, and the primary analysis was limited to patients with complete longitudinal data. Biological mechanisms, including inflammatory markers, autonomic function, immune parameters, and histological changes, were not directly measured. Therefore, the proposed trajectory-based framework should be regarded as hypothesis-generating rather than as an established classification system or validated clinical decision-making tool. Future prospective studies using independent cohorts, standardized EAT protocols, validated outcome definitions, and biological measurements are needed to confirm the reproducibility, clinical utility, and mechanistic significance of these temporal response phenotypes.

## References

[1] Horiguchi S (1966). Epipharyngitis and its relationship to general diseases (in Japanese). J Jpn Soc Head Neck Surg.

[2] Harabuti Y (2025). Nasopharyngeal swabbing therapy for chronic nasopharyngitis (in Japanese). J Jpn Soc Otolaryngol Head and Neck Surg.

[3] Tanaka A (2018). Studies on band-limited light endoscopic diagnosis and endoscopic epipharyngeal abrasive therapy in chronic epipharyngitis (in Japanese). Stomato-Pharyngology.

[4] Hotta O, Inoue C, Tanaka A, Ieiri N (2017). Possible mechanisms underlying epipharyngeal abrasive therapy (EAT) with ZnCl2 solution for the treatment of autoimmune diseases and functional somatic syndrome. J Antivir Antiretrovir.

[5] Nishi K, Yoshimoto S, Tanaka T (2025). Spatial transcriptomics of the epipharynx in long COVID identifies SARS-CoV-2 signalling pathways and the therapeutic potential of epipharyngeal abrasive therapy. Sci Rep.

[6] Hotta O, Tanaka A, Oda T (2019). Chronic epipharyngitis: a missing background of IgA nephropathy. Autoimmun Rev.

[7] Hirobumi I (2024). Autonomic stimulation action of EAT (epipharyngeal abrasive therapy) on chronic epipharyngitis. Cureus.

[8] Hirobumi I (2025). Autonomic reflexes with epipharyngeal abrasive therapy (EAT). Cureus.

[9] Hirobumi I (2026). Longitudinal response to epipharyngeal abrasive therapy (EAT) in chronic nasopharyngitis with outcome redefinition and stratified analysis by tissue type and baseline severity. Cureus.

[10] Boessen R, Groenwold RH, Knol MJ, Grobbee DE, Roes KC (2012). Classifying responders and non-responders; does it help when there is evidence of differentially responding patient groups?. J Psychiatr Res.

[11] Tamm J, Takano K, Just L, Ehring T, Rosenkranz T, Kopf-Beck J (2026). Early improvement predicts treatment response in depression: an ecological momentary assessment study in an inpatient and day clinic setting. Behav Ther.

[12] Bhidayasiri R, Koebis M, Kamei T, Ishida T, Suzuki I, Cho JW, Wu SL (2023). Sustained response in early responders to safinamide in patients with Parkinson's disease and motor fluctuations: a post hoc analysis of the SETTLE study. Front Neurol.

[13] Hirobumi I (2025). Epipharyngeal abrasive therapy (EAT) field theory: a clinical framework for EAT in modulating psycho-neuro-endocrino-immune (PNEI) dynamics. Cureus.

[14] Ito H (2022). Statistical study of the effectiveness of EAT treatment for chronic epipharyngitis. Schol J Otolaryngol.

[15] Ohno Y (2022). Treatment outcome for chronic epipharyngitis using the evaluation method of the Epipharyngeal Abrasive Therapy Review Committee (in Japanese). Stomato-Pharyngology.

[16] Mogitate M (2023). Epipharynegal abrasive therapy downregulates the number of epipharyngeal abrasive CD4 cells with symptomatic recovery. Cureus.

